# Relatedness of Gardnerella prophages and their endolysins

**DOI:** 10.64898/2026.09.28.754664

**Published:** 2026-10-10

**Authors:** Brandon S. Maust, Kira A. Griswold, Monique Khim, Sandhya Subramanian, Bart Staker, Peter J. Myler, Brian R. Kullin, Anna-Ursula Happel, Arvind Varsani, Heather B. Jaspan

## Abstract

The Gardnerella genus includes a complex sub-taxonomy of bacterial species and subgroups which have been implicated in bacterial vaginosis. We analyzed G. leopoldii, G. swidsinski, G. piotii, and G. vaginalis genomes available in public databases and predicted proviral sequences using Cenote-Taker3. We clustered 266 prophage sequences into 140 viral genera level groupings that encode four distinct gene clusters and 19 different lysin proteins. Analysis of the bacterial and prophage sequence phylogenies reveals that while there were no specific prophage sequence lineages that infected each bacterial host, there is clustering of the prophage sequences by bacterial species within the Gardnerella genus.

## Full Text Availability

The license terms selected by the author(s) for this preprint version do not permit archiving in PMC. The full text is available from the preprint server.

